# Biomimetic Chromatography as a High-Throughput Tool for Screening Bioaccumulation and Acute Aquatic Toxicity of Pesticides

**DOI:** 10.3390/jox16010004

**Published:** 2025-12-26

**Authors:** Krzesimir Ciura

**Affiliations:** Department of Physical Chemistry, Faculty of Pharmacy, Medical University of Gdansk, Aleja Generała Józefa Hallera 107, 80-416 Gdansk, Poland; krzesimir.ciura@gumed.edu.pl

**Keywords:** IAM chromatography, phospholipophilicity, bioaccumulation, aquatic toxicity, fish LC_50_, *Daphnia magna*, pesticides

## Abstract

Modern pesticide risk assessment relies on data on bioaccumulation and acute aquatic toxicity, yet generating such data is labour-intensive and animal-demanding. This study evaluated whether phospholipid affinity of pesticides, quantified by the chromatographic hydrophobicity index CHI_IAM_ obtained from high-throughput gradient biomimetic chromatography, can serve as a surrogate descriptor of these endpoints. Nineteen pesticides representing different chemical and functional classes were analyzed on IAM.PC.DD2 columns, and CHI_IAM_ values were determined. Bioconcentration factors (BCF) in fish and acute toxicity data (96 h LC_50_ for fish, 48 h EC_50_ for *Daphnia magna*) were retrieved from the Pesticide Properties DataBase. CHI_IAM_ ranged from −12.1 to 54.8 and correlated strongly with log_10_BCF (r = 0.84) and log_10_LC_50_ in fish (r = −0.84), and moderately with log_10_EC_50_ for *Daphnia* (r = 0.76). Highly lipophilic pesticides with high CHI_IAM_ showed elevated BCF and low LC_50_/EC_50_ values, whereas polar compounds with low CHI_IAM_ exhibited negligible bioconcentration and low acute toxicity. Deviations from these trends, for compounds with specific modes of action, highlighted the contribution of mechanisms beyond membrane toxicity. Overall, CHI_IAM_ measured under high-throughput conditions retains prognostic value for ecotoxicological assessment and may serve as a rapid experimental descriptor to support preliminary screening.

## 1. Introduction

Modern agriculture relies on the routine and systematic use of numerous plant protection products to maintain and improve crop productivity [1]. As a consequence, pesticides, together with other biologically active contaminants such as antibiotics and hormones, are now among the most frequently detected pollutants in aquatic and terrestrial ecosystems [2,3,4]. These compounds may pose significant risks to non-target organisms [5]. In regulatory environmental risk assessment of plant protection products, substance characterization typically begins with key physicochemical properties such as water solubility, vapour pressure and volatility, dissociation constants, and octanol–water partitioning, which are then integrated with environmental fate and behaviour data including degradation and photolysis in soil and in water–sediment systems, as well as adsorption–desorption related mobility. In the aquatic compartment, core ecotoxicological endpoints include acute toxicity to fish and aquatic invertebrates, reported as 96 h LC_50_ values for fish and 48 h EC_50_ values for *Daphnia magna*, together with effects on primary producers such as algal growth inhibition and tests with aquatic plants, and where relevant chronic and sediment-dweller studies as well as the bioconcentration factor (BCF) [6,7,8,9]. These parameters are used within regulatory frameworks to identify chemicals of concern. Nevertheless, generating experimental toxicity and bioaccumulation data for the growing number of active substances is labour-intensive, costly and heavily dependent on animal testing [10]. Consequently, there is a strong incentive to develop alternative in vitro and in silico methods capable of predicting bioaccumulation and aquatic toxicity and thereby supporting environmental risk assessment [11].

Among the various molecular properties that influence these endpoints, lipophilicity has long been recognized as a key determinant of bioaccumulation and baseline toxicity in aquatic organisms [12]. Traditionally, the octanol–water partition coefficient (logKow, often denoted as logP in medicinal chemistry) has served as a surrogate descriptor of a chemical’s lipophilicity and its potential to bioaccumulate and it also strongly affects the distribution of pesticides between the aqueous phase, suspended particulate matter and organic matrices, with important consequences for their extraction efficiency, recovery and apparent stability in analytical determinations [2,4]. Numerous studies have reported apparent linear relationships between logKow and log BCF [13]. However, these simple models exhibit well-known limitations. In particular, highly hydrophobic compounds often deviate from the expected linear trend and display lower bioaccumulation than predicted, for example due to reduced bioavailability, sorption to particulate matter or enhanced biotransformation [14]. From an experimental standpoint, the classical shake-flask determination of logKow is also relatively labour-intensive, time-consuming and costly, which further motivates the search for more efficient and biorelevant approaches to characterizing lipophilicity.

One such approach is biomimetic chromatography, originally introduced by Valkó and co-workers in pharmaceutical research for the rapid characterization of bio-physicochemical properties of drug candidates [15,16,17]. Biomimetic chromatographic systems employ stationary phases coated with biomolecules to mimic key biological interfaces. Among them, immobilized artificial membrane (IAM) chromatography has played a particularly important role. In IAM stationary phases, phosphatidylcholine is covalently bonded to a silica support to form a monolayer that mimics the polar–apolar interface of a cell membrane. Chromatographic indices derived from IAM retention, such as phospholipid affinity parameters obtained under isocratic or gradient conditions, have been successfully used to describe membrane interactions and to support predictions of pharmacokinetic behaviour and nonspecific toxicity.

Several studies have highlighted the potential of IAM chromatography and related systems in environmental and ecotoxicological applications. Fernández-Pumarega et al. compared conventional reversed-phase chromatography, IAM chromatography and micellar electrokinetic chromatography to mimic nonspecific aquatic toxicity and soil sorption of selected organic compounds [18]. Tsopelas et al. demonstrated that IAM retention factors for pharmaceuticals correlate with BCF values in fish, enabling improved bioconcentration predictions for ionizable and polar compounds that are often mispredicted by logKow based models [19]. Stergiopoulos and co-workers showed that IAM retention data for pesticides can be linearly related to acute toxicity values across multiple aquatic species, including fish and *Daphnia*, and that these relationships frequently outperform models relying solely on logKow [14]. In subsequent studies, micellar chromatographic systems were also shown to predict aquatic toxicity of pesticides reasonably well, albeit with slightly lower performance compared with IAM [20]. Furthermore, gradient IAM protocols and chromatographic hydrophobicity indices (CHI_IAM_) have been applied to drugs and UV filters to support the prediction of their aquatic toxicity [21].

Despite these advances, the systematic evaluation of structurally diverse pesticides using high-throughput gradient IAM protocols and their direct linkage to standard ecotoxicological endpoints, such as 96 h LC_50_ for fish, 48 h EC_50_ for *Daphnia magna* and BCF in fish, remains limited. The working hypothesis of the present study is that the phospholipid affinity of pesticides, quantified by IAM chromatography, constitutes a major determinant of their bioaccumulative behaviour and acute aquatic toxicity. To address this hypothesis, gradient IAM chromatography was applied to a set of 19 pesticides from different chemical and functional classes, including insecticides, herbicides and fungicides. Using literature-derived ecotoxicological parameters, the ability of IAM-derived descriptors to capture variability in bioaccumulation and acute toxicity across species was assessed, and the usefulness of IAM chromatography as a potential screening tool to support environmental risk assessment of pesticides was evaluated.

## 2. Materials and Methods

### 2.1. Analytes

A set of 19 pesticides was selected to cover a broad range of chemical classes and lipophilicities. The compounds included organophosphate and carbamate insecticides (azinphos-methyl, chlorpyrifos, diazinon, fenitrothion, carbaryl), pyrethroid insecticides (bifenthrin, fenpropathrin, permethrin), neonicotinoid insecticides (imidacloprid, acetamiprid), triazine herbicides (atrazine, terbuthylazine), other herbicides (dicamba, imazapyr) and fungicides (azoxystrobin, boscalid, fludioxonil, propiconazole, tebuconazole). Analytical standards were purchased from Supelco (fenitrothion; Steinheim, Germany), VWR Chemicals (azinphos-methyl, chlorpyrifos, diazinon; Radnor, PA, USA), and Sigma-Aldrich (carbaryl, bifenthrin, fenpropathrin, permethrin, imidacloprid, acetamiprid, atrazine, terbuthylazine, dicamba, imazapyr, azoxystrobin, boscalid, fludioxonil, propiconazole, tebuconazole; Steinheim, Germany).

### 2.2. Biochromatographic Analysis

All biochromatographic experiments were performed using the gradient IAM protocols developed by Valkó and co-workers and previously implemented in this laboratory. Analyses were carried out on a Prominence-i LC-2030C 3D HPLC system (Shimadzu, Tokyo, Japan) controlled by LabSolutions software (v6.81, Kyoto, Japan) and equipped with an IAM.PC.DD2 column (10 × 4.6 mm, 10.0 µm) packed on the newer ‘Type B’ silica support and protected by a guard column (Regis Technologies, Morton Grove, IL, USA).

Mobile phase A consisted of 50 mM ammonium acetate (VWR International, Leuven, Belgium) in water, adjusted to physiological pH 7.4 with concentrated ammonia solution (Avantor Performance Materials Poland S.A., Gliwice, Poland). Ultrapure water with a resistivity of 18.2 MΩ was obtained from a Milli-Q water purification system (Merck Millipore, Darmstadt, Germany). For phospholipid affinity measurements, mobile phase B was HPLC-grade acetonitrile (Chempur, Piekary Śląskie, Poland). A linear gradient from 0 to 85% B was applied over 5.25 min, followed by an isocratic hold at 85% B for 0.5 min. The mobile phase flow rate was set at 1.5 mL/min, and the column temperature was maintained at 30 °C.

The reference substances used to calculate CHI_IAM_ values were obtained as follows: acetanilide, butyrophenone and octanophenone from Alfa Aesar (Haverhill, MA, USA); acetophenone and paracetamol from Sigma-Aldrich (Steinheim, Germany); and heptanophenone, hexanophenone, propiophenone and valerophenone from Acros Organics (Pittsburgh, PA, USA).

Dimethyl sulfoxide (Avantor Performance Materials Poland S.A., Gliwice, Poland) was used to dissolve the analytes and to prepare stock solutions at a concentration of 200 µg/mL. UV detection was performed in the range 190–300 nm. The injection volume was 5 µL. Each compound was analyzed in duplicate, and the variation in retention times was lower than 2%. Raw retention times for the pesticides and reference standards are available in Zenodo platform https://doi.org/10.5281/zenodo.17672582.

### 2.3. Toxicity Data

Aquatic toxicity and bioaccumulation data for the pesticides were obtained from the Pesticide Properties DataBase (PPDB; University of Hertfordshire). Three endpoints were considered: (i) 96 h LC_50_ values for rainbow trout (*Oncorhynchus mykiss*), expressed in mg/L (LC_50_ fish); (ii) 48 h EC_50_ values for *Daphnia magna* (mg/L); and (iii) bioconcentration factors (BCF) in fish (dimensionless). The values for each pesticide are summarized in Table 1.

## 3. Results and Discussion

Previous studies have demonstrated that IAM chromatography is a powerful tool for linking phospholipid affinity with bioaccumulation and baseline toxicity of various xenobiotics [22,23], including bisphenols [24], UV filters [21], pharmaceuticals [19,23] and selected pesticides [14]. These investigations have provided both local models for structurally related compounds and more global models covering a chemically diverse library of substances [25]. However, most of the available data were generated using earlier IAM stationary phases, such as IAM.MG.PP, which are no longer commercially available. Additionally, analyses were usually performed under isocratic conditions, which do not provide high-throughput capability.

In this context, the present study was designed to evaluate whether IAM indices obtained with a high-throughput gradient protocol on IAM.PC.DD2 columns can be successfully applied to ecotoxicological profiling of pesticides. To achieve this goal, a structurally diverse set of 19 pesticides was analyzed, and the resulting CHI_IAM_ values were related to standard aquatic toxicity and bioaccumulation endpoints.

The obtained CHI_IAM_ values are presented in Table 1 and span a very wide range, reflecting substantial differences in phospholipid affinity among tested pesticides. The most hydrophilic compound in the set was the herbicide imazapyr, which showed a negative CHI_IAM_ (−12.1). Such a negative value indicates that imazapyr eluted almost immediately, essentially before any significant amount of organic modifier was added; this is consistent with its highly polar, anionic nature and low lipophilicity, leading to minimal retention on the phospholipid column. In contrast, the highest CHI_IAM_ values were observed for hydrophobic insecticides such as permethrin (54.8) and bifenthrin (54.4), belonging to the pyrethroid family. Many other insecticides (e.g., chlorpyrifos, fenpropathrin) and certain hydrophobic fungicides (e.g., fludioxonil) also showed high CHI_IAM_ values in the 40 s to 50 s. Moderately lipophilic compounds, including atrazine, carbaryl, boscalid and the triazole fungicides (propiconazole, tebuconazole), exhibited intermediate or high CHI_IAM_ values in the range of approximately 30–50. Meanwhile, polar or moderately polar pesticides such as the neonicotinoids (imidacloprid, CHI_IAM_ 14.2; acetamiprid, 13.4) and the herbicide dicamba (8.2) had low CHI_IAM_ values, signifying low phospholipid affinity consistent with their high water solubility. These results confirm that CHI_IAM_ successfully captures the relative lipophilicity of the pesticides in a biologically relevant, membrane-mimetic context.

Similarly to the wide range of experimentally determined phospholipid affinities, the selected pesticides exhibited a broad spectrum of toxicity towards aquatic organisms. Fish LC_50_ values covered almost five orders of magnitude, from highly toxic pyrethroid insecticides such as bifenthrin (LC_50_ ≈ 0.00026 mg/L) and fenpropathrin (≈0.0023 mg/L) to practically non-toxic herbicides such as imidacloprid, acetamiprid and imazapyr (LC_50_ ≥ 83–100 mg/L). *Daphnia magna* 48 h EC_50_ values revealed an even more pronounced sensitivity range: several insecticides were extraordinarily toxic, with chlorpyrifos and diazinon showing EC_50_ values around 0.0001–0.001 mg/L (sub-ppb levels), whereas herbicides such as atrazine and imazapyr were much less toxic (EC_50_ ≈ 85 mg/L and ≈100 mg/L, respectively), presumably due to their plant-specific mode of action and low bioavailability to the animals.

**Figure 1:**
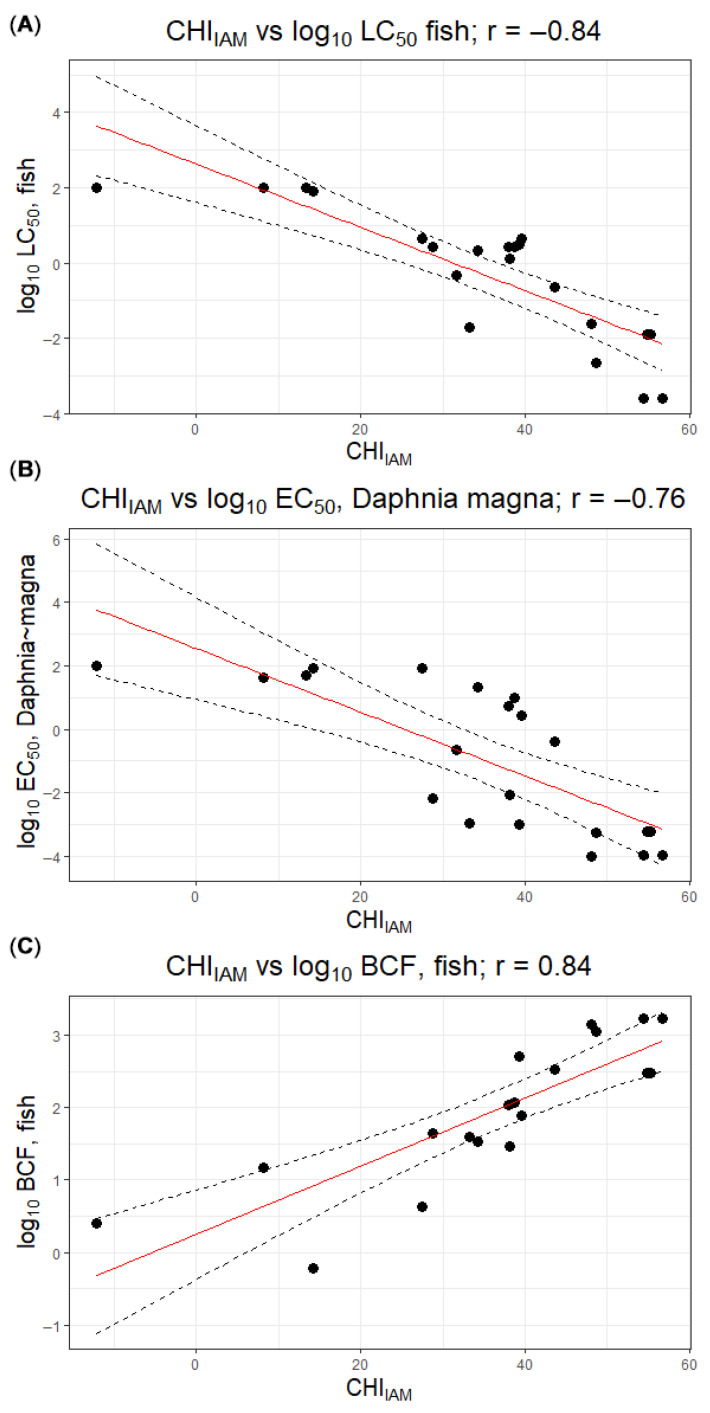
Relationships between CHI_IAM_ and log_10_-transformed ecotoxicological endpoints for the studied pesticides: (**A**) log_10_LC_50_, fish, (**B**) log_10_EC_50_, *Daphnia magna*, (**C**) log_10_BCF, fish. Points represent experimental data, and dashed lines indicate linear regression fits with their 95% confidence limits. The solid red line marks the line of identity (y = x), corresponding to perfect agreement.

Fish bioconcentration factors (BCFs) also varied widely and generally reflected lipophilicity. Pesticides with low CHI_IAM_ values (e.g., imidacloprid, CHI_IAM_ 14.2) showed negligible bioconcentration (BCF ≈ 0.6, effectively no accumulation in fish tissues), whereas highly lipophilic insecticides such as bifenthrin and chlorpyrifos (CHI_IAM_ > 48) had BCF values on the order of 10^3^ (1703 and 1374, respectively), indicating strong bioaccumulation. Intermediate BCFs were observed for moderately hydrophobic compounds (e.g., boscalid, CHI_IAM_ 38.0, BCF ≈ 107; terbuthylazine, CHI_IAM_ 34.3, BCF ≈ 34). Overall, the data collated in Table 1 illustrate clear qualitative links between membrane affinity (CHIIAM), bioconcentration potential and acute aquatic toxicity. These relationships were further quantified by calculating Pearson correlation coefficients between CHI_IAM_ and log_10_-transformed endpoints: strong correlations were observed for fish acute toxicity (r = −0.84 for CHI_IAM_ vs. log_10_LC_50_), *Daphnia magna* toxicity (r = −0.76 for CHI_IAM_ vs. log_10_EC_50_) and bioaccumulation (r = 0.84 for CHI_IAM_ vs. log_10_BCF), with higher membrane affinity being associated with lower LC_50_/EC_50_ values and higher BCF (Figure 1).

These findings are consistent with the concept of baseline toxicity being largely driven by membrane partitioning: hydrophobic organic compounds accumulate in cell membranes, and lethal effects often occur when a critical membrane concentration is reached. In the present data set, the most toxic chemicals to both fish and *Daphnia*, such as bifenthrin, fenpropathrin, chlorpyrifos and azinphos-methyl, combine high CHI_IAM_ values with large BCFs, reflecting their pronounced bioaccumulative and toxic potential. A few deviations from this simple pattern merit mention. Permethrin, for example, has an extremely high CHI_IAM_ (54.8) and is very toxic to fish (LC_50_ ≈ 0.01 mg/L), but its measured BCF (≈300) is lower than that of bifenthrin despite comparable CHI_IAM_, which can plausibly be attributed to faster biotransformation or elimination. Conversely, carbaryl, a moderately hydrophobic carbamate (CHI_IAM_ ≈ 28.8), is far more toxic to *Daphnia* (EC_50_ ≈ 0.0064 mg/L) than would be predicted from lipophilicity alone, reflecting its specific neurotoxic mode of action in aquatic invertebrates.

The weaker correlation observed between CHI_IAM_ and *Daphnia* EC_50_ can be rationalized by physiological differences between fish and cladocerans. In fish, the presence of a blood–brain barrier means that neurotoxic effects often depend on a compound’s ability to cross this barrier, a process strongly influenced by lipophilicity. In *Daphnia*, the absence of such protective barriers and the open circulatory system allow even relatively hydrophilic toxicants to access neural tissues more directly, reducing the dependence of toxicity on membrane partitioning. As a result, specific modes of action can produce extreme sensitivity irrespective of CHI_IAM_, leading to outliers and an overall weaker CHI_IAM_–toxicity relationship.

Nevertheless, the results of this study provide clear evidence that a pesticide’s affinity for phospholipid membranes, as quantified by IAM chromatography, is an important determinant of its environmental bioaccumulation and acute toxicity profile. Numerous environmental monitoring studies have shown that pesticides are among the most frequently detected micropollutants in aquatic ecosystems, which underlines the need for efficient tools to prioritize substances for more detailed ecotoxicological and fate assessments [2,3,4].

One practical implication is therefore the potential use of IAM chromatography as part of an integrated, tiered strategy for environmental risk screening and chemical prioritization. Because CHI_IAM_ can be generated rapidly using short analytical runs, IAM provides a high-throughput, experimentally grounded descriptor that can be combined with conventional physicochemical and fate information to support grouping, ranking, and weight-of-evidence reasoning at early tiers. This direction is consistent with the broader transition in chemical safety assessment towards new approach methodologies, NAMs, and integrated approaches that aim to increase mechanistic interpretability while adhering to the principles of replacement, reduction and refinement of animal testing [26]. 

Importantly, the role and decision value of routine vertebrate testing in aquatic risk assessment is actively being re-examined, including acute fish toxicity testing for pesticides, in view of both scientific utility and 3R considerations. Within this landscape, rapid non-animal experimental descriptors that help identify substances most likely to drive risk can support more targeted deployment of resource-intensive guideline studies [27,28,29].

## 4. Conclusions

The present study shows that the phospholipid affinity of pesticides, quantified by the IAM-based index CHI_IAM_, is closely related to their bioaccumulation potential and acute aquatic toxicity (LC_50_, EC_50_, BCF). Despite several mode-of-action-driven outliers, higher CHI_IAM_ values were generally associated with stronger bioconcentration and lower effect concentrations, supporting the role of membrane partitioning in baseline toxicity. CHI_IAM_ determined with a short high-throughput gradient on IAM.PC.DD2 columns can therefore be considered a useful experimental descriptor for preliminary ecotoxicological screening and prioritization of pesticides.

## Figures and Tables

**Table 1:** Overview of 96 h LC_50_ (fish, mg/L), 48 h EC_50_ (*Daphnia magna*, mg/L), bioconcentration factors (BCF) and IAM chromatographic hydrophobicity indices (CHI_IAM_) for the studied pesticides.

Pesticide	LC_50_ Fish	EC_50_ *Daphnia*	BCF	CHI_IAM_
Imidacloprid	83	85	0.61	14.2
Acetamiprid	100	49.8	N.A.	13.4
Atrazine	4.5	85	4.3	27.4
Azinphos-methyl	0.02	0.0011	40	33.2
Azoxystrobin	0.47	0.23	N.A.	31.6
Bifenthrin	0.00026	0.00011	1703	54.4
Boscalid	2.7	5.33	107	38.0
Carbaryl	2.6	0.0064	44	28.80
Chlorpyrifos	0.025	0.0001	1374	48.1
Diazinon	3.1	0.0010	500	39.3
Dicamba	100	41	15	8.2
Fenitrothion	1.3	0.0086	29	38.1
Fenpropathrin	0.0023	0.00053	1100	48.6
Fludioxonil	0.23	0.4	336	43.5
Imazapyr	100	100	2.54	−12.1
Permethrin	0.0125	0.0006	300	54.8
Propiconazole	2.6	10.2	116	38.7
Tebuconazole	4.4	2.79	78	39.5
Terbuthylazine	2.2	21.2	34	34.3

N.A.—data not available.

## Data Availability

The data presented in this study are openly available in Zenodo platform at [https://doi.org/10.5281/zenodo.17672582].

## Abbreviations

BCF	fish bioconcentration factors
CHI_IAM_	chromatographic index of hydrophobicity of IAM
IAM	immobilized artificial membrane
